# Molecular characterization of a new microvariant of the G3 genotype for *Echinococcus granulosus* in water buffalo in Iran

**Published:** 2015-03-15

**Authors:** Marzie Babazadeh, Hassan Sharifiyazdi, Mohammad Moazeni, Sedigheh Gorjipour, Mahdi Heidari

**Affiliations:** 1 *Department of Clinical Sciences, School of Veterinary Medicine, Shiraz University, Shiraz, Iran; *; 2 *Department of Pathobiology, School of Veterinary Medicine, Shiraz University, Shiraz, Iran.*

**Keywords:** *Echinococcus granulosus*, G3 genotype, Iran, Mitochondrial gene, Water buffalo

## Abstract

In this study, molecular characterization of *Echinococcus granulosus* sample obtained from water buffalo originating from southwest of Iran was performed using comparative sequence analysis of *cox*1 mitochondrial gene. DNA was extracted from protoscoleces removed from hydatid cyst from the liver of a 2-year-old male buffalo slaughtered in Khuzestan province. Molecular and phylogenetic analyses were conducted based on* cox*1 mitochondrial gene. We found the presence of a new microvariant of G3 genotype for *E. granulosus *in Iran which is genetically differentiated from reference G3 sequence (M84663). The difference was a transition mutation of adenine to guanine in position 214 (A214G) resulting in a substitution of the threonine (ACT) by alanine (GCT). These findings extend the knowledge of heterogeneity and distribution of G3 genotype for *E. granulosus* in world.

## Introduction

*Echinococcosis* infection is a cosmopolitan zoonosis caused by the adult or larval stages of cestodes belonging to the genus *Echinococcus* (family: Taeniidae). The disease is one of the more prevalent infections in Iran especially in rural areas, where offal from abattoirs is improperly disposed, or where slaughtering is done on farms.^1^ The parasite has an indirect life cycle. Adult worms occur in dogs and other canids as definitive hosts and many herbivorous and omnivorous species, including sheep, cattle, camels, pigs and humans as intermediate hosts.^2^ Hydatidosis in buffaloes is an important disease and leads to significant financial losses from condemnation of edible offal, milk, skin and energy in draught power and is also transmissible to humans.^3^ Various survey reported the prevalence of hydatid cyst in slaughtered buffaloes from 0.89% to 57.76% in different parts of Iran.^4^^-^^8^

 To date, ten distinct genotypes (G1-G10) of *Echinococcus granulosus* have been recognized.^9^^,^^10^ According to recent taxonomic revisions the* E. granulosus* has been ordered into 4 species namely* E. granulosus* sensu stricto (G1-G3), *E. equines *(G4),* E. ortleppi *(G5) and* E. canadensis *(G6-G10).^11^ The water buffalo is mainly susceptible to the G3 strain, although the sheep strain G1 and cattle strain G5 can infect this animal.^12^ *Echinococcus* *granulosus* strains could have important implications for host specificity, antigenicity, transmission dynamics, infection route, pathology, control, antimicrobial susceptibility, life-cycle patterns of parasite, developmental rates, fertility of developed cysts, biochemistry, infectivity in human, diagnostic reagents and vaccine development strategies.^13^ Previous study mainly described the morphological and molecular characterization of *E*. *granulosus* isolates from sheep, goat, cattle, camel and human in different parts of Iran including north, center and south,^14^ however, there is limited molecular study dealing with the hydatid cyst from water buffaloes, in Khuzestan province, southwest Iran. This paper described a new microvariant of the G3 genotype for *E. granulosus* obtained from buffalo originating from southwest of Iran using comparative sequence analysis of *cox*1 mitochondrial gene. 

## Materials and Methods

Hydatid cyst was collected from liver of a 2-year-old male buffalo slaughtered in Khuzestan province, south-west Iran. Cyst contents were examined under light microscopy for the presence of protoscoleces. The collected protoscoleces were rinsed five times with sterile phosphate buffer saline (PBS) and then fixed and preserved in 70% (v/v) ethanol until DNA isolation. Following removal of the ethanol, DNA was extracted from protoscoleces using a commercial DNA extraction kit (DNesasy; Qiagen, Valencia, USA) according to the manufacturer’s protocol.

The mitochondrial DNA was amplified using specific primers for Cytochrome C oxidase 1 (*cox*1) JB3/JB4 (5´-TTT TTT GGG CAT CCT GAG GTT TAT -3´/5´-TAA AGA AAG AAC ATA ATG AAA ATG-3´).^15^ The PCR was performed in a thermocycler with the following program: an initial denaturation step at 95 ˚C for 5 min, 35 cycles of denaturation at 94 ˚C for 1 min, annealing at 55 ˚C for 1 min, and extension at 72 ˚C for 1 min, followed by a final extension at 72 ˚C for 5 min. The amplified products were purified with a PCR product purification kit (Bioneer, Alameda, USA) and sequenced directly using the capillary DNA analyzer (Model ABI 3730; Applied Biosystems, Foster City, USA). Nucleotide sequence analysis was undertaken using the national center for biotechnology information BLAST (Basic Local Alignment Search Tool) programs and databases.^16^ The different genotypes of *E. granulosus*, G1-G10 were used for phylogenetic analysis. Multiple sequence alignments and construction of a phylogenetic tree were made with the maximum-parsimony method using the MEGA software (Version 4.0; Biodesign Institute, Tempe, USA).^17^

## Results

**Fig. 1:**
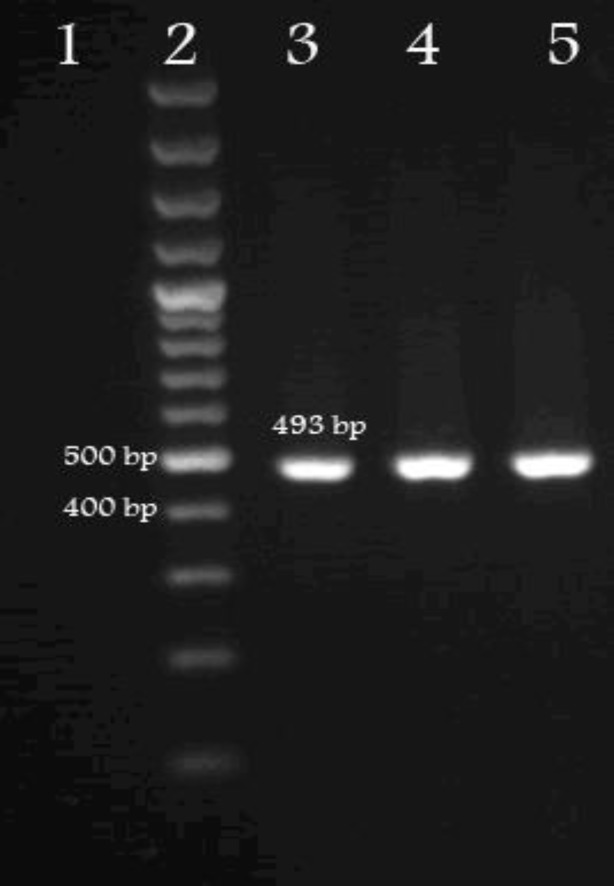
Electrophoresis analysis of *cox*1 (493 bp). PCR amplification provided from water buffalo sample (Lane 3 and 4: duplicate samples were used for sequencing) compared with the molecular weight marker (Lane 2: 100 bp) and positive control (Lane 5). Lane 1 is negative control.

Microscopically, cyst was found to be fertile and PCR was positive by producing a *cox*1 fragment from DNA of *E. granulosus* as shown in Figure 1. 

**Fig. 2:**
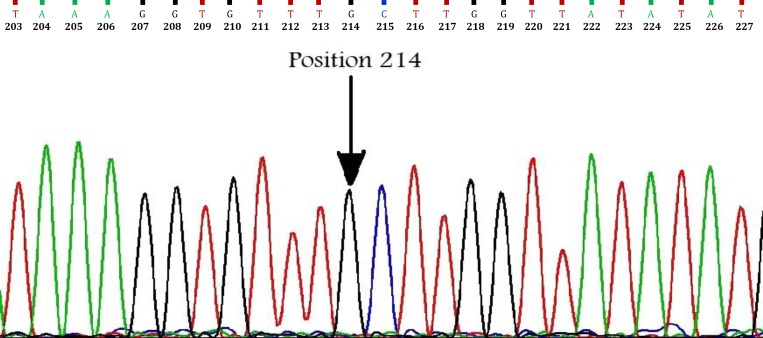
Partial chromatogram of the cox1 sequence. Dark arrow showing sense mutation of adenine to guanine in position 214.

**Fig. 3:**
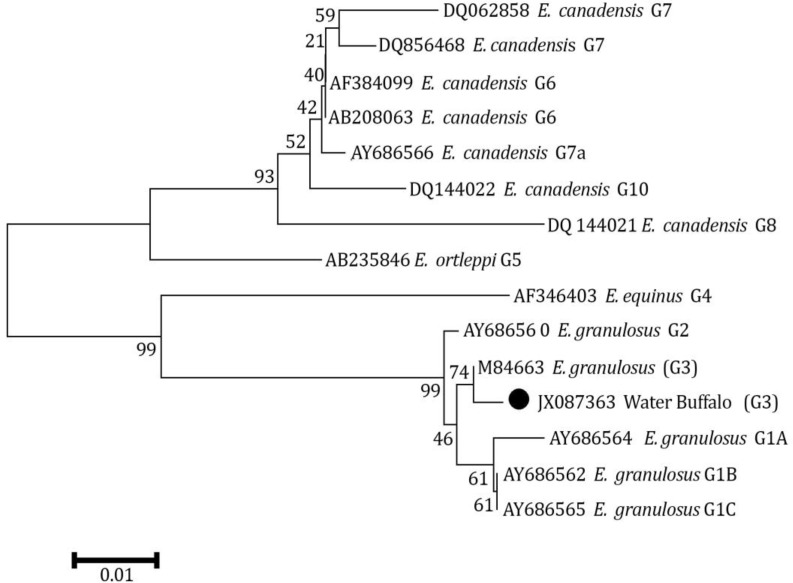
A phylogenetic tree of *Echinococcus granulosus* genotypes inferred from the nucleotide sequences of partial *cox*1 and the maximum-parsimony method. Numerals indicate bootstrap values (%) from 1,000 replicates.

*Echinococcus granulosus* isolate described here was found to have complete identity with the *cox*1 fragment previously described by Bowles *et al*. for G3 reference sequence except at one nucleotide position.^14^ The difference was a transition mutation of adenine to guanine in position 214 (A214G) resulting in a substitution of the threonine (ACT) by alanine (GCT) according to the echinoderm mitochondria genetic code (Fig. 2). Also, phylogenetic analysis of concatenated data showed this isolate was grouped into a distinct cluster corresponding to the G1-G3 complex with the most closely relation to G3 genotype (Fig. 3).

## Discussion

According to the latest available data, there are about 460 thousand of buffaloes in Iran with approximately 138 thousand of them in the southwest region.^18^ Hydatidosis is an important zoonotic disease that constitutes a major public health in many countries throughout the world such as Iran.^19^ The application of molecular tools to the characterization of the etiological agents of echinococcosis has revealed a series of largely host-adapted species and genotypes that are maintained in distinct cycles of transmission.^20^ Camels and buffalo were found to have the highest prevalence of hydatid cysts. This is most likely due to the older age at which the animals are slaughtered.^21^ In order to develop preventive and control strategies for echinococcosis, a better knowledge of transmission cycle of *E. granulosus* complex is necessary. Intraspecific variants have been described for *E*. *granulosus* complex from different species of intermediate hosts in different geographical areas and several strains.^20^

Some investigators in various parts of the world have carried out molecular studies of the parasite. Molecular techniques have validated the genetic basis of important morphological differences that can now be used with confidence as a reliable and simple means of identifying and differentiating between strains and species of *Echinococcus*.^22^ The genotypes are also important regarding the host specificity and life cycle of the *E. granulosus*.^23^^,^^24^ In the current study we used a mitochondrial marker for phylogenetic studies and population differentiation because of its relatively rapid rate of evolution, importance in differentiation and discrimination of closely related geno-types (i.e. G2 and G3). It is maternally inherited and does not undergo any recombination.^11^^,^^25^^-^^28^ For the first time we showed the presence of the G3 genotype in water buffalo in southwest of Iran. Recently, Amin-Pour *et al*. reported two isolates of G3 genotypes which have 100% identity to reference G3 strain (M84663) in buffaloes generating from West Azerbaijan, where this province has common border with east and southeast of Turkey.^26^ They could not found any G3 genotypes among buffalo samples (n = 3) obtained from southwest of Iran. Interestingly, Vural *et al*. have also reported G3 strain from buffaloes in east and southeast of Turkey.^28^ Moreover, G3 genotype was first recovered in camel in center of Iran.^29^ Also, this strain was found in sheep and cattle in Italy.^30^ 

According to the *cox*1 gene, our G3 nucleotide sequence (JX087363) differed from G3 nucleotide reference sequence (M84662), in one nucleotide at position 214 (Fig. 2). Although the sequences reported by Amin-Pour *et al*. and Sharbatkhori *et al*. for the Iranian G3 genotype are completely homologous to the reference sequence of G3 (M84663),^26^^,^^29^^,^^31^^,^^32^ a silent mutation (C to T at position 168) was detected in the *cox*1 sequences of the Iranian G3 camel isolates (HM626405) reported by Sharifiyazdi *et al*.^27^

More recently, Parsa *et al*. reported G3 sequences in dogs from Iran that showed 100% homology with G3 reference sequence in *nad*1 (AJ237633), but displayed two different *cox*1 profiles, each having 99.00% homology with reference G3 sequence (M84663).^33^ However, our study indicated that the Khuzestan strain of G3 is genetically differentiated from other G3 isolates reported so far from different geographic regions of the world. It is assumed that *E*. *granulosus* was most likely introduced into southwest Iran from Iraq through livestock transportation. Thus, additional analyses for specimens collected from neighboring countries of Iran are needed to evaluate how various G3 genotypes has been introduced and spread in Iran.

In Tunisia, M'rad *et al*. showed a mutation of a cytosine to a thymine in position 44 (C44T) resulting in a substitution of the alanine in position 15 by a valine according to the echinoderm mitochondrial genetic code on G3.^34^ In addition, Beyhan and Umur reported that six out of *E. granulosus* isolates from water buffalo in Turkey belonged to G1 genotypes while other samples showed variant genotypes of G1-G2-G3 complex.^35^ Two of them showed a thymine in position 52, and one isolate showed a single nucleotide change compared to strain G1 (C122T). It seems that all of these mutations introduce new genotypes in the *E. granulosus* population and may lead to the adaptation of populations to their local environmental conditions. The investigation described here extended the information about the distribution of G3 genotype of *E. granulosus* in Iran and identified a new microvariant of this genotype which could be important for hydatid control and public health.

## References

[1] Mooraki A, Rahbar MH, Bastani B (1996). Spontaneous systemic anaphylaxis as an unusual presentation of hydatid cyst: Report of two cases. Am J Trop Med Hyg.

[2] Vural G, Baca AU, Gauci CG (2008). Variability in the Echinococcus granulosus Cytochrome C oxidase 1 mitochondrial gene sequence from livestock in Turkey and a re-appraisal of the G1-3 genotype cluster. Vet Parasitol.

[3] Shamsul Islam AWM (1982). Hydatidosis in buffaloes in Bangladesh. Rev Sci Tech Off Int Epiz.

[4] Dadkhah MA, Yeganehzad M, Naderi B (2011). Survey on hydatid cyst infestation in Sarab city (Northwest of Iran) using epidemiological and sero-epidemiological criteria. J Anim Vet Adv.

[5] Daryani A, Alaei R, Arab R (2007). The prevalence, intensity and viability of hydatid cysts in slaughtered animals in the Ardabil province of northwest Iran. J Helminthol.

[6] Khanmohammadi M, Maghami SG, Zakariazadeh M (2008). The prevalence of hydatidosis by sex, season and location in slaughtered buffaloes at the Tabriz abattoir in 2006-2007. Int J Vet Med.

[7] Rahimi MT, Sharifdini M, Ahmadi A (2011). Hydatidosis in human and slaughtered herbivores in Mazandaran, northern Iran. Asian Pac J Trop Dis.

[8] Sarmast MH, Javaherizadeh H, Hojati M (2011). Hydatid cyst disease in Khuzestan province, Iran. East Cent Afr J Surg.

[9] Romig T (2003). Epidemiology of echinococcosis. Langenbecks Arch Surg.

[10] Nakao M, McManus DP, Schantz PM (2007). A molecular phylogeny of the genus Echinococcus inferred from complete mitochondrial genomes. Parasitology.

[11] McManus DP, Thompson RCA (2003). Molecular epidemiology of cystic echinococcosis. Parasitology.

[12] Thompson RCA, McManus DP (2002). Towards a taxonomic revision of the genus Echinococcus. Trends Parasitol.

[13] Thompson RCA, McManus DP, Eckert J, Gemmell MA, Meslin FX (2001). Etiology: Parasites and life-cycles. WHO/OIE manual on echinococcosis in human and animals.

[14] Hosseini SH, Eslami A (1998). Morphological and developmental characteristics of Echinococcus granulosus derived from sheep, cattle and camels in Iran. J Helminthol.

[15] Bowles J, Blair D, McManus DP (1992). Genetic variants within the genus Echinococcus identified by mitochondrial DNA sequencing. Mol Biochem Parasitol.

[16] BLAST: Basic local alignment search tool.

[17] Tamura K, Dudley J, Nei M (2007). MEGA4: Molecular evolutionary genetics analysis (MEGA) software version 4. Mol Biol Evol.

[18] Taheri-Dezfuli B, Nejati-Javaremi A, Abbasi MA (2011). Economic weights of milk production traits for buffalo herds in the southwest of Iran using profit equation. World Appl Sci J.

[19] Shahnazi M, Hejazi H, Salehi M (2010). Molecular characterization of human and animal Echinococcus granulosus isolates in Isfahan, Iran. Acta Trop.

[20] Thompson R (2008). The taxonomy, phylogeny and transmission of Echinococcus. Exp Parasitol.

[21] Latif AA, Tanveer A, Maqbool A (2010). Morphological and molecular characterization of Echinococcus granulosus in livestock and humans in Punjab, Pakistan. Vet Parasitol.

[22] Thompson RCA, Lymbery AJ, Baker JR (1988). The nature, extent and significance of variation within the genus Echinococcus. Adv Parasitol.

[23] Bowles J, McManus DP (1993). Molecular variation in Echinococcus. Acta Trop.

[24] Dinkel A, Njoroge EM, Zimmermann A (2004). A PCR system for detection of species and genotypes of the Echinococcus granulosus complex, with reference to the epidemiological situation in eastern Africa. Int J Parasitol.

[25] Ahmadi N, Dalimi A (2006). Characterization of Echinococcus granulosus isolates from human, sheep and camel in Iran. Inf Gen Evol.

[26] Amin-Pour A, Hosseini S, Shayan P (2011). Comparative geno-typing of Echinococcus granulosus infecting buffalo in Iran using cox1 gene. Parasitol Res.

[27] Sharifiyazdi H, Oryan A, Ahmadnia S (2011). Genotypic characterization of Iranian camel (Camelus dromedarius) isolates of Echinoccocus granulosus. J Parasitol.

[28] Vural G, Baca AU, Gauci CG (2008). Variability in the Echinococcus granulosus cytochrome C oxidase 1 mitochondrial gene sequence from livestock in Turkey and a re-appraisal of the G1 genotype cluster. Vet Parasitol.

[29] Sharbatkhori M, Fasihi-Harandi M, Mirhendi H (2011). Sequence analysis of cox1 and nad1 genes in Echinococcus granulosus G3 genotype in camels (Camelus dromedarius) from central Iran. Parasitol Res.

[30] Busi M, Snabel V, De Liberato C (2004). Molecular genotyping of Echinococcus granulosus hydatid cysts in Italy reveals the presence of three distinct genotypes. Parasitologia.

[31] Sharbatkhori M, Mirhendi H, Jex AR (2009). Genetic categorization of Echinococcus granulosus from humans and herbivorous hosts in Iran using an integrated mutation scanning-phylogenetic approach. Electrophoresis.

[32] Sharbatkhori M, Mirhendi H, Fasihi-Harandi M (2010). Echinococcus granulosus genotypes in livestock of Iran indicating high frequency of G1 genotype in camels. Exp Parasitol.

[33] Parsa F, Fasihi-Harandi M, Rostami S (2012). Genotyping Echinococcus granulosus from dogs from western Iran. Exp Parasitol.

[34] M'rad S, Oudni M, Filisetti D (2010). Molecular identification of Echinococcus granulosus in Tunisia: First record of the buffalo strain (G3) in human and bovine in the country. Open Vet Sci J.

[35] Beyhan YE, Umur S (2011). Molecular characterization and prevalence of cystic echinococcosis in slaughtered water buffaloes in Turkey. Vet Parasitol.

